# Integrating bulk and single-cell RNA sequencing identifies and validates lactylation-related signatures in diabetic foot ulcers

**DOI:** 10.1038/s41598-026-49753-z

**Published:** 2026-04-27

**Authors:** Xiao Peng, Wenqiang Wang, Xinyu Nie, Qikai Hua

**Affiliations:** 1 The First Affiliated Hospital of Guangxi Medical University, Nanning, Guangxi Zhuang Autonomous China; 2 Department of Orthopedics, The First Affiliated Hospital of the University of Science and Technology of China, No. 17, Lujiang Road, Hefei City, Anhui Province China

**Keywords:** Diabetic foot ulcer, lactylation, single-cell RNA sequencing, machine learning, fibroblast heterogeneity, metabolic reprogramming, immune microenvironment, Biomarkers, Cell biology, Computational biology and bioinformatics, Diseases, Immunology

## Abstract

Diabetic foot ulcers (DFU) is severe complications of diabetes mellitus, affecting 15–25% of diabetic patients and imposing substantial healthcare burdens. Lactylation has emerged as a critical regulator in inflammation and wound healing processes. However, the role of lactylation-related mechanisms in DFU pathogenesis remains poorly understood. We integrated the lactylation-related genes (LRGs) and systematically analysed the expression levels of these genes to characterize a gene signature related to DFUs through bulk RNA-seq and single-cell RNA-seq (scRNA-seq) datasets. Single-cell RNA sequencing identified ten major cell populations in DFU tissues. Augur prioritization and AUCell analysis revealed significantly elevated lactylation-related gene (LRG) activity in fibroblasts and multiple immune-related cell types in DFU, with fibroblasts exhibiting the strongest transcriptional perturbation. Subclustering analysis defined a DFU-enriched fibroblast subset (DFU_Fibroblasts), in which hdWGCNA identified red and brown gene modules tightly associated with this population. Bulk RNA-seq integration identified six differentially expressed LRGs enriched in mitochondrial respiratory chain organization and pyruvate metabolism, suggesting metabolic remodeling. Machine learning approaches further identified four hub LRGs (*USB1*, *COX5A*, *LDHA*, and *NFU1*) with robust diagnostic performance (AUC = 0.904 in the training cohort). Immune infiltration analysis revealed altered macrophage polarization and NK cell imbalance in DFU. At the single-cell level, hub LRG–high fibroblasts displayed glycolysis-oriented metabolic features, early pseudotime positioning, and enhanced outgoing signaling toward monocytes via the *PPIA–BSG* and *ANGPTL2–ITGA5 + ITGB1* axes. Molecular docking indicated favorable binding between capsaicin and *COX5A* and *LDHA*, and qPCR validation confirmed significant upregulation of all four hub genes in DFU samples. Our study identifies a lactate-associated transcriptional program in DFU that is linked to fibroblast heterogeneity, metabolic reprogramming, and alterations in the immune microenvironment. The identified hub LRGs (*COX5A*, *LDHA*, *USB1* and *NFU1*) exhibit diagnostic potential and may represent candidate molecular targets within this metabolic–inflammatory network. These findings provide a mechanistic framework for understanding the involvement of lactylation-related pathways in DFU progression and support further investigation into targeted therapeutic strategies.

**Supplementary Information:**

The online version contains supplementary material available at 10.1038/s41598-026-49753-z.

## Introduction

Diabetic foot ulcers (DFU) represent one of the most severe complications of diabetes mellitus, affecting approximately 15–25% of diabetic patients during their lifetime^1^. These chronic wounds not only impose a substantial economic burden on healthcare systems but also significantly impair patients’ quality of life and frequently lead to lower limb amputations^2^. The pathophysiology of DFUs is complex and multifactorial, involving peripheral neuropathy, vascular insufficiency, altered biomechanics, and impaired wound healing processes^3^.

Recent advances in metabolic research have uncovered novel post-translational modifications that regulate cellular function, among which lactylation has emerged as a critical regulator in various pathological conditions^4^. Unlike traditional histone modifications, lactylation, derived from intracellular lactate accumulation, modulates gene expression through the modification of histones and non-histone proteins^5^. This metabolic-epigenetic crosstalk has been implicated in inflammation, wound healing, and tissue repair processes. The accumulation of lactate in diabetic tissues results from metabolic reprogramming characterized by enhanced glycolysis and compromised oxidative phosphorylation^6^. This metabolic shift not only provides energy for cellular function but also serves as a source for lactylation modifications that may influence gene expression and cellular fate decisions. Understanding the role of lactylation-related mechanisms in DFU pathogenesis could provide new therapeutic targets for this devastating complication.

Single-cell RNA sequencing (scRNA-seq) has revolutionized our understanding of cellular heterogeneity in disease states, allowing for the identification of specific cell populations and their transcriptional states^7^. In the context of DFUs, fibroblasts have been identified as key players in the impaired wound healing process, exhibiting distinct metabolic and transcriptional signatures compared to normal skin tissue^8^. The application of advanced computational methods, including machine learning algorithms and network analysis, has further enhanced our ability to identify disease-relevant molecular patterns and potential therapeutic targets^9^.

In this study, we integrate bulk RNA-seq and scRNA-seq datasets to comprehensively characterize lactylation-related signatures in DFU tissues. By employing multiple machine learning algorithms (LASSO, SVM-RFE, Random Forest, GBM, and Boruta) and advanced single-cell analysis techniques, we aim to identify key lactylation-related genes (LRGs) that contribute to DFU pathogenesis. Furthermore, we investigate the cellular heterogeneity, immune microenvironment alterations, and metabolic reprogramming patterns associated with these LRGs, with particular emphasis on fibroblast subpopulations. Our findings not only provide mechanistic insights into the role of lactylation in DFU but also identify potential therapeutic targets and validate their drugability through molecular docking analysis.

## Methods

### Data acquisition and processing

The scRNA-seq dataset GSE165816, comprising 14 DFU foot-skin samples and 19 non-ulcer foot-skin control samples (11 healthy and 8 diabetic without DFU), was downloaded from the Gene Expression Omnibus (GEO) database (http://www.ncbi.nlm.nih.gov/geo)^8^. Only foot-skin specimens were included in the analysis, whereas forearm skin and PBMC samples were excluded to avoid tissue-source heterogeneity (Supplementary Table 1). In addition, four bulk RNA sequencing datasets were obtained for machine learning analyses to enhance the robustness and generalizability of the study: GSE80178 and GSE134431 were used as the training cohort, whereas GSE199939 and GSE68183 served as validation cohor^10^ t. The detailed characteristics of these bulk datasets, including platform information, technology type, sample size, tissue source, and analytical role, are summarized in Table 1. A total of 334 LRGs were selected based on previous studies^10–13^.

**Table 1:** Characteristics of bulk transcriptomic datasets included in this study.

Dataset	Platform (GPL)	Technology	DFU (*n*)	Control (*n*)	Tissue
GSE68183	GPL16686	Microarray	3	3	Foot skin
GSE80178	GPL16686	Microarray	6	6	Foot skin
GSE134431	GPL18573	RNA-seq	13	8	Foot skin
GSE199939	GPL24676	RNA-seq	10	11	Foot skin
GSE68183	GPL16686	Microarray	3	3	Foot skin

### ScRNA-seq analysis

Single-cell data preprocessing and analysis were conducted using the R package “Seurat” (v4.1.1)^7^. Cells were filtered to retain high-quality data: Cells with > 50% mitochondrial gene expression were excluded; Cells with > 1% erythrocyte gene expression were excluded; Cells expressing fewer than 200 or more than 2500 genes were removed^14,15^. Potential doublets were identified and removed using the DoubletFinder algorithm prior to normalization and clustering analysis^16^. Batch effects among samples were corrected using the “Harmony” package, and data normalization was performed using the ScaleData function. Principal Component Analysis (PCA) was applied to identify significant components, which were further used for Uniform Manifold Approximation and Projection (UMAP) dimensionality reduction through the RunUMAP function. Cell clustering was conducted using the FindClusters function, and marker genes for each cluster were identified using FindAllMarkers. Cell type annotation was carried out with the “SingleR” package^17^.

To assess the activity levels of LRG gene sets in single cells, the AUCell algorithm was applied. Here, LRG activity was defined as a gene signature score rather than pathway enrichment. AUCell calculates the area under the cumulative distribution curve (AUC) of gene expression ranks to score gene set activity in each individual cell, thereby quantifying the relative enrichment of the predefined LRG signature at the single-cell level^18^. Single-cell Weighted Gene Co-expression Network Analysis (hdWGCNA) was performed to identify gene co-expression modules. Module eigengenes were computed using the harmonized module eigengene (hME) approach at the metacell level, and module activity was subsequently quantified for downstream analyses^19^. Modules represent clusters of genes with similar expression patterns, and intramodular connectivity reflects the strength of interactions within modules, aiding in hub gene identification. The Augur algorithm was employed to prioritize cell types exhibiting significant transcriptomic perturbations across different biological states^20^. Cell-cell communication analysis was conducted using the CellChat package to infer ligand-receptor interaction networks between cell populations^21^. Single-cell developmental trajectory inference was performed using Monocle2 and CytoTRACE to explore lineage differentiation paths^22^.

To investigate metabolic reprogramming at the single-cell level, the scMetabolism R package was employed. Predefined metabolic gene sets were used to calculate metabolic activity scores in each cell via the AUCell method embedded within scMetabolism^23^.

### Machine learning-based construction of an DFU-related predictive model

Five independent machine learning approaches were applied to identify robust LRG candidates associated with DFU, including Boruta, LASSO regression, SVM-RFE, Random Forest, and Gradient Boosting Machine. LASSO feature selection was performed under a binomial framework with 10-fold cross-validation to determine the optimal penalty parameter (λ_min). Random Forest models were constructed using 1,000 trees, and feature importance was ranked based on Gini impurity. GBM models were trained with 5-fold cross-validation using standard boosting parameters. SVM-RFE was conducted with a linear kernel to iteratively remove low-weight features. Boruta analysis was performed with 500 iterations to confirm all relevant variables.

Genes consistently selected across all five algorithms were defined as hub LRGs using an intersection strategy. The discriminative performance of these hub genes was evaluated by receiver operating characteristic (ROC) analysis, and their differential expression was further validated in independent external datasets.

### Functional enrichment analysis

Gene Ontology (GO) analysis is a widely used method for large-scale functional enrichment, encompassing biological processes (BP), molecular functions (MF), and cellular components (CC). The Kyoto Encyclopedia of Genes and Genomes (KEGG) is an extensively utilized database that provides information on genomes, biological pathways, diseases, and drugs. Differentially expressed genes (DEGs) between the groups were subjected to GO annotation and KEGG pathway enrichment analysis using the clusterProfiler package in R. A p-value < 0.05 was considered statistically significant^24^.

To investigate key biological processes associated with hub genes, we performed Gene Set Enrichment Analysis (GSEA), which assesses whether specific gene sets exhibit statistically significant differences between the groups^25^. For GSEA, we used the ranked differential expression results derived from the single-cell comparison between Fibroblasts_high and Fibroblasts_low. Genes were ranked by a weighted score incorporating the direction of log2 fold change and statistical significance (sign(log2FC) × −log10(p-value)). Enrichment analysis was performed against the KEGG pathway database (not Hallmark), using permutation-based GSEA (nPerm = 10,000; minGSSize = 5). Multiple testing was corrected using the Benjamini–Hochberg method, and pathways with FDR/q value < 0.05 were considered significantly enriched.

Additionally, to compare the differences in related biological processes between the groups, Gene Set Variation Analysis (GSVA) was conducted^26^. GSVA was performed using the “c2.cp.kegg.symbols.gmt” gene set collection from MSigDB. Enrichment scores were computed using the GSVA R package (ssGSEA/GSVA framework), and group-wise differences in pathway scores were assessed using two-sided t-tests. P values were adjusted using the Benjamini–Hochberg FDR method, and pathways with p.adjust < 0.05 were considered statistically significant.

### Analysis of the immune microenvironment

Immune infiltration analysis was performed using the algorithm based on 22 immune cell signatures to assess the immune landscape between DFU and control samples^27^. The relative abundance of immune cells was visualized using bar plots, and group differences were compared using the Wilcoxon rank-sum test and displayed as boxplots. The correlations between hub LRGs expression and immune infiltration levels were assessed via Spearman correlation, and significant associations were visualized using lollipop plots and correlation heatmaps.

### Identification of LRGs subtypes associated with DFU

Unsupervised consensus clustering was performed using the “ConsensusClusterPlus” package to explore potential subtypes of DFU based on LRGs expression profiles^28^. Parameters included: maximum number of clusters (maxK = 9), partitioning around medoids (PAM) clustering algorithm, Euclidean distance metric, and 50 resampling iterations. The optimal cluster number was determined based on cumulative distribution function (CDF) plots and area under the CDF curve. PCA was conducted to verify clustering robustness and visualize the distribution of the identified subtypes.

### Molecular docking of key LRGs with small molecules

Potential therapeutic compounds targeting the hub LRGs were identified through Enrichr by querying the DSigDB v1.0 database. The 3D protein structures of the key LRGs were retrieved from the Protein Data Bank. Molecular docking simulations were performed using AutoDock4 and AutoGrid4, with docking scores lower than − 5.0 kcal/mol considered indicative of strong binding affinity. Visualization of docking poses and ligand-receptor interactions was conducted using PyMOL^29^.

### qPCR detection

DFU patient and normal control skin tissue samples (*n* = 6 each) were collected, and total RNA was extracted using TRIzol reagent, followed by reverse transcription to cDNA using a reverse transcription kit. qPCR reactions were performed on an ABI 7500 Real-Time PCR System with a reaction mixture containing SYBR Green Master Mix, cDNA template, and specific primers. The primer sequences for the four target genes were: USB1-F: TGACCTCCTTCCACAGGTGC, USB1-R: ATCTTGTCACCCGGTCCAC; COX5A-F: AGGCTTAGGGGACTGGTTGT, COX5A-R: GGAACACACGGTAAGAGGGC; LDHA-F: AACTGCAAACTCCAAGCTGGT, LDHA-R: TCTTTCCAAGCCACGTAGGTC; NFU1-F: TGACGCAGTAGCCTGCAAAC, NFU1-R: TGCCTAAGGGTCTCCCTGAC. PCR cycling conditions consisted of initial denaturation at 95 °C for 10 min, followed by 40 cycles of denaturation at 95 °C for 15 s and annealing/extension at 60 °C for 1 min. GAPDH was used as the internal reference gene, and relative expression levels were calculated using the 2^-ΔΔCt method with three technical replicates for each sample.

## Results

### Single-cell analysis

**Fig. 1:**
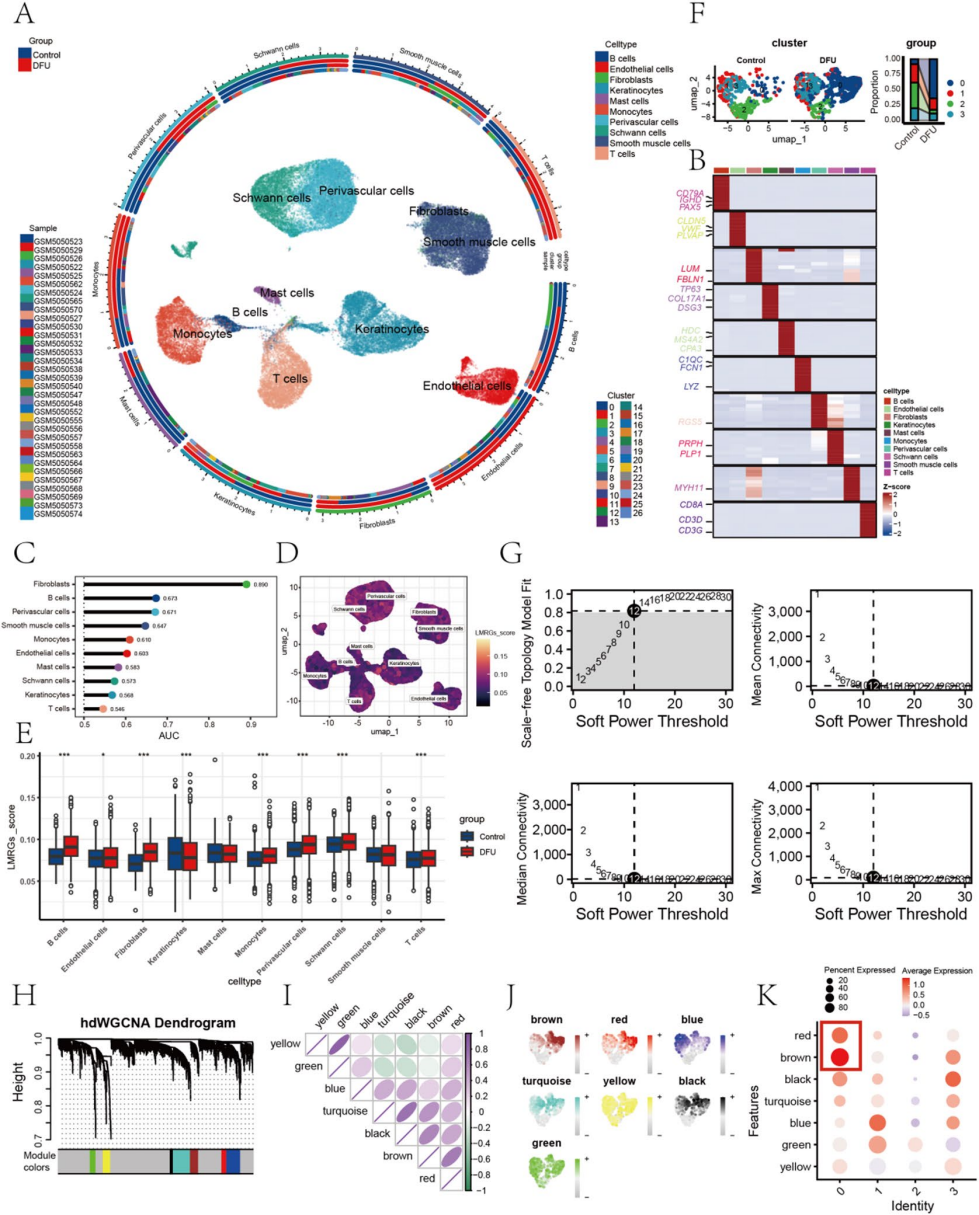
Integrated scRNA-seq analysis reveals the cellular heterogeneity of control and DFU skin tissues. (**A**) Single-cell landscape visualized by UMAP. (**B**) Expression patterns of representative marker genes across cell types. (**C**) Prioritization of cluster-specific gene expression differences using Augur. (**D**) UMAP visualization of LRG activity scores. (**E**) Box plots comparing LRG scores between the control and DFU groups. (**F**) UMAP visualization and relative proportions of fibroblast subclusters in the Control and DFU groups. (**G**) Soft threshold selection for hdWGCNA. (**H**) Dendrogram of gene clustering showing 12 identified modules. (**I**) Correlation matrix among different gene modules. (**J**) UMAP visualization of module eigengenes. (**K**) Dot plot showing module activity across cell types. Statistical significance was defined as *p < 0.05, **p < 0.01, ***p < 0.001 unless otherwise indicated.

A comprehensive overview of quality control for our scRNA-seq data (GSE165816), is presented in Fig. 1A. Cells were retained according to the following criteria: 200–2,500 detected genes per cell (Supplementary Fig. 1), mitochondrial gene expression < 50% (percent.mt), and erythrocyte-related gene expression < 1%. Doublet detection was performed using the DoubletFinder algorithm prior to downstream analyses. After quality control filtering and doublet removal, high-quality cells were retained for subsequent analysis. Unsupervised clustering of the integrated scRNA-seq dataset identified ten transcriptionally distinct cell populations (Fig. 1A). Based on canonical marker gene expression (Fig. 1B), these clusters were annotated as B cells (CD79A, IGHD, PAX5), endothelial cells (CLDN5, VWF, PLVAP), fibroblasts (LUM, FBLN1), keratinocytes (TP63, COL17A1, DSG3), mast cells (CPA3, MS4A2, HDC), monocytes (LYZ, FCN1, C1QC), perivascular cells (RGS5), Schwann cells (PRPH, PLP1), smooth muscle cells (MYH11), and T cells (CD8A, CD3D, CD3G). We next evaluated cell-type-specific gene expression alterations between control and DFU skin tissues using the Augur algorithm (Fig. 1C). Augur prioritizes cell types based on their classification performance (AUC) between experimental conditions, thereby quantifying the degree of condition-associated transcriptional perturbation. Fibroblasts exhibited the highest Augur score among all identified cell types. Subsequently, we utilized the AUCell R package to investigate LRG activity across different cell types, aiming to characterize the expression features of LRGs (Fig. 1D). Comparative analysis demonstrated that LRG activity was significantly upregulated in perivascular cells (*P* < 0.001), fibroblasts (*P* < 0.001), B cells (*P* < 0.001), monocytes (*P* < 0.001), Schwann cells (*P* < 0.001), and T cells (*P* < 0.001) in the DFU group compared with controls. A modest increase was also observed in endothelial cells (*P* = 0.041). In contrast, LRG activity was significantly decreased in keratinocytes (*P* < 0.001) (Fig. 1E). Notably, fibroblasts simultaneously displayed the highest Augur prioritization score and elevated LRG activity, supporting their prominent transcriptional responsiveness in DFU. These findings suggest that fibroblasts may play a crucial role in LRG-mediated DFU progression. Functional enrichment analysis was performed across all identified cell clusters (Supplementary Fig. 2). Notably, enrichment analysis specific to fibroblasts highlighted significant involvement in pathways related to protein secretion and extracellular protein localization, suggesting enhanced secretory activity. In addition, terms associated with positive regulation of the ERK1/2 cascade and regulation of lymphocyte proliferation were enriched, indicating potential roles in MAPK pathway activation and immune modulation. Given the pivotal role of fibroblasts in LRG-mediated DFU progression, we focused our subsequent analyses on this cell population. Unsupervised clustering of fibroblasts identified four distinct subclusters. Notably, cluster 0 was predominantly enriched in the DFU group (Fig. 1F), suggesting a potential disease-associated phenotype. Accordingly, cluster 0 was designated as DFU_Fibroblasts. To further characterize the transcriptional programs underlying this subset, we performed hdWGCNA analysis to identify potential fibroblast-specific hub genes. Using a soft-thresholding power of 12, seven gene modules were identified (Fig. 1G-H). Among these, the red and brown modules were strongly positively correlated, indicating coordinated module activity (Fig. 1I). Importantly, both UMAP visualization and bubble plot analysis consistently demonstrated substantial overlap between the red and brown modules and cluster 0 fibroblasts (Fig. 1J-K), supporting the robustness of the module–cluster association. Therefore, the top 30 genes from the red and brown modules were defined as hub genes of DFU_Fibroblasts (Supplementary Table 2).

### Identification of DEGs and enrichment analysis

**Fig. 2:**
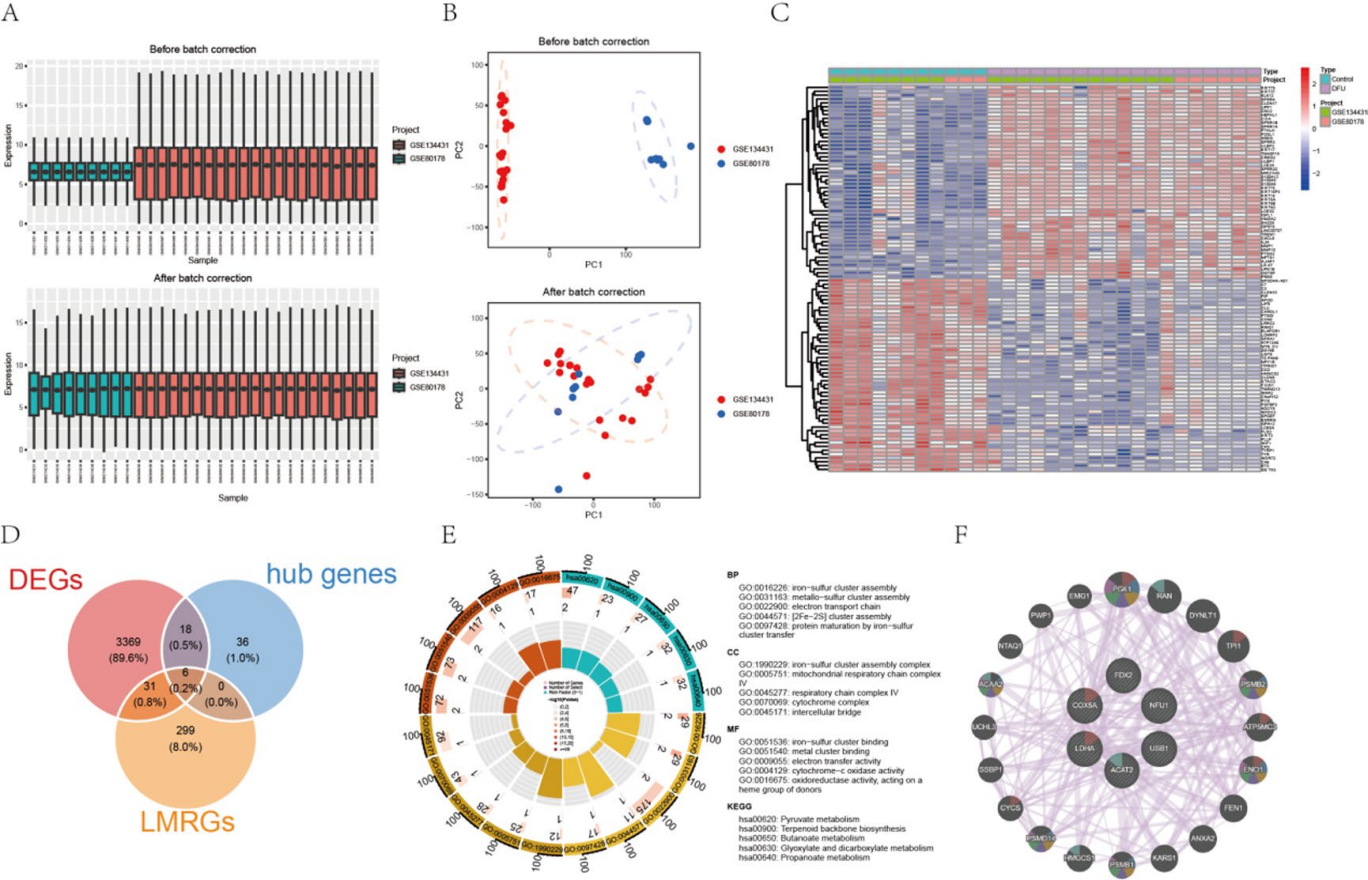
Identification and enrichment analysis of DEGs in DFU. (**A–B**) Boxplots and PCA plots illustrating batch effects before and after correction. (**C**) Heatmap displaying the expression profiles of top DEGs. (**D**) Venn diagram identifying 7 overlapping DE-LRGs. (**E**) GO and KEGG pathway enrichment analysis of DE-LRGs. (**F**) GeneMANIA network showing functional enrichment and interaction analysis of DE-LRGs.

We next focused on identifying DEGs associated with DFU to explore potential molecular mechanisms. Two bulk RNA-seq datasets (GSE80178 and GSE134431) containing 10 control samples and 20 DFU samples were obtained from the GEO database. Batch effects were corrected using the “SVA” package, and data normalization was performed. PCA demonstrated that significant batch effects existed before correction, while samples clustered more tightly after correction, indicating effective removal of batch effects and suitability for downstream analysis (Figs. 2A–B). Subsequently, differential expression analysis was conducted using the “limma” package, with selection criteria set at |log₂ fold change| > 0.585 and p-value < 0.05. A total of 3,423 DEGs were identified, comprising 1,591 upregulated and 1,832 downregulated genes. Heatmaps were generated to visualize the distribution and expression patterns of DEGs (Figs. 2C). A Venn diagram was used to visualize the overlap among DEGs, LRGs, and hub genes of DFU_Fibroblasts, resulting in the identification of six overlapping differentially expressed LRGs (DE-LRGs), namely USB1, COX5A, ACAT2, NFU1, LDHA, and FDX2 (Fig. 2D). GO enrichment analysis demonstrated that DE-LRGs were predominantly associated with iron–sulfur cluster assembly, mitochondrial respiratory chain organization, and electron transport processes. KEGG pathway analysis further highlighted enrichment in pyruvate metabolism, suggesting altered metabolic flux between glycolysis and mitochondrial respiration (Fig. 2E). GeneMANIA network analysis revealed that DE-LRGs and their interacting partners were closely connected to ATP metabolic processes and cellular responses to hypoxia (Fig. 2F), indicating coordinated regulation of energy production and oxygen-sensitive metabolic adaptation. Collectively, these findings suggest that LRG-associated transcriptional alterations in DFU are characterized by a shift toward glycolysis-linked metabolic reprogramming, mitochondrial functional remodeling, and hypoxia-responsive signaling, thereby providing a metabolic basis for lactate accumulation and potential lactylation-mediated gene regulation.

### Identification of LRGs

**Fig. 3:**
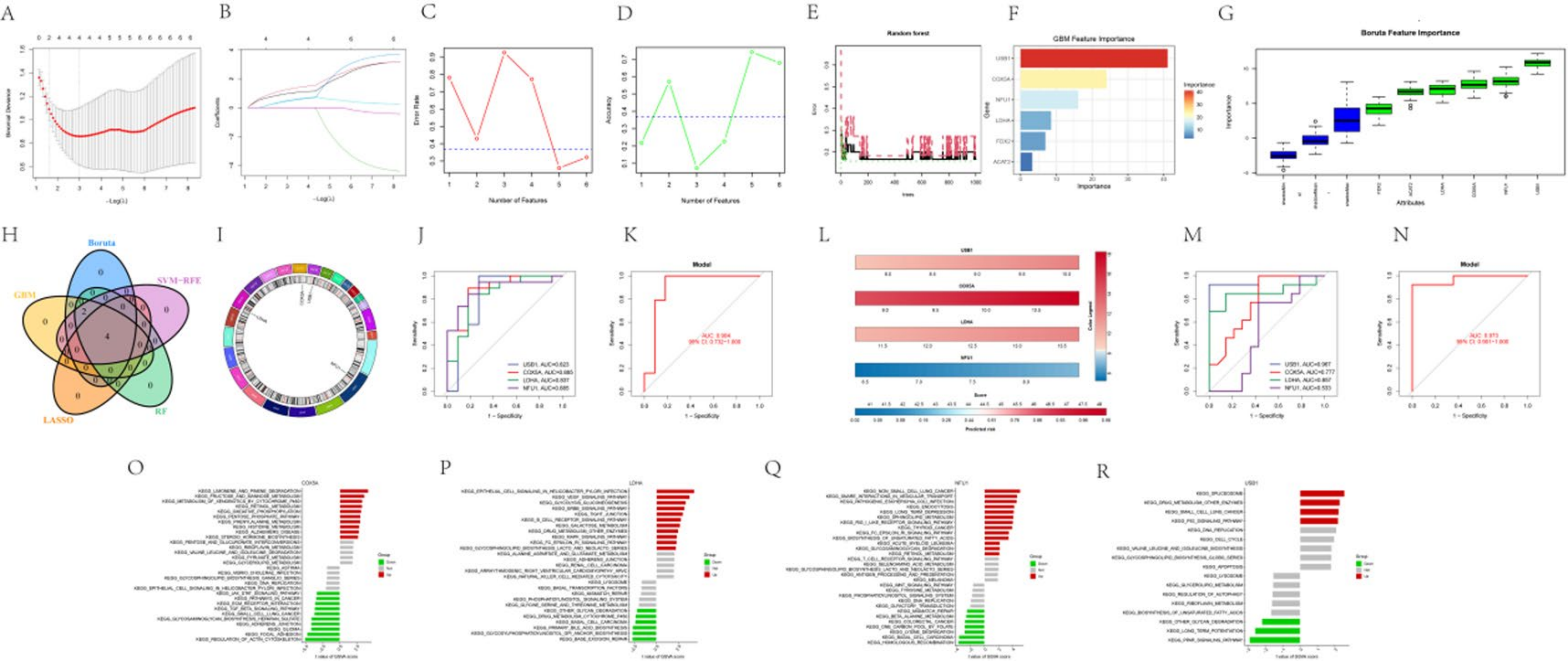
Machine learning-based identification and functional characterization of lactylation-related hub genes. (**A–B**) LASSO regression screening of candidate DE-LRGs. (**C–D**) SVM-RFE screening results. (**E**) Feature gene selection based on Random Forest model. (**F**) Feature gene selection based on GBM model. (**G**) Feature gene selection using Boruta algorithm. (**H**) Venn diagram showing four overlapping genes selected by five machine learning methods. (**I**) Chromosomal distribution of the four hub genes. (**J**) ROC curves of *USB1*, *COX5A*, *LDHA*, and *NFU1* in the training cohort. (**K**) ROC curve of the nomogram model constructed from four hub genes. (**L**) Calibration curve of the nomogram model. (**M**) ROC curves of *USB1*, *COX5A*, *LDHA*, and *NFU1* in the external validation cohort. (**N**) ROC curve of the validation cohort. (**O–R**) GSVA analysis showing the pathway associations of the four hub genes.

To identify key LRGs involved in DFU, five machine learning algorithms were applied, including LASSO, SVM-RFE, RF, GBM, and Boruta. Feature selection was independently performed by each algorithm to screen for the most relevant candidate genes in the training cohort (Figs. 3A–G). By integrating the results of all five algorithms, four overlapping feature genes—*USB1*, *COX5A*, *LDHA*, and *NFU1*—were identified (Fig. 3H). The chromosomal locations of these four genes were further visualized (Fig. 3I). ROC curve analysis demonstrated strong diagnostic performance for these genes, with AUC values of 0.823, 0.885, 0.837, and 0.885 for *USB1*, *COX5A*, *LDHA*, and *NFU1*, respectively, in the training cohort (Fig. 3J). A nomogram model based on these four genes was subsequently constructed, achieving an AUC of 0.904 in the training cohort (Figs. 3K–L). Validation in an independent cohort integrating GSE199939 and GSE68183 (13 DFU and 14 control samples) confirmed the diagnostic performance of the four hub LRGs, as demonstrated by favorable ROC curves (Figs. 3M–N). GSVA further explored the biological functions associated with the hub LRGs (Figs. 3O–R). *COX5A* was predominantly enriched in oxidative phosphorylation and mitochondrial energy metabolism pathways. *LDHA* was strongly associated with glycolysis/gluconeogenesis and MAPK signaling, consistent with metabolic reprogramming and stress-responsive signaling. In contrast, *NFU1* and *USB1* were enriched in DNA replication, mismatch repair, and cell cycle–related pathways, suggesting activation of genomic maintenance programs under metabolic perturbation. Collectively, these findings indicate that the four hub LRGs converge on mitochondrial function, glycolytic remodeling, and stress-adaptive regulatory pathways, supporting their potential involvement in lactate-associated metabolic dysregulation in DFU.

### Immune infiltration, molecular subtypes, and functional characterization of hub LRG-related patterns in DFU

**Fig. 4:**
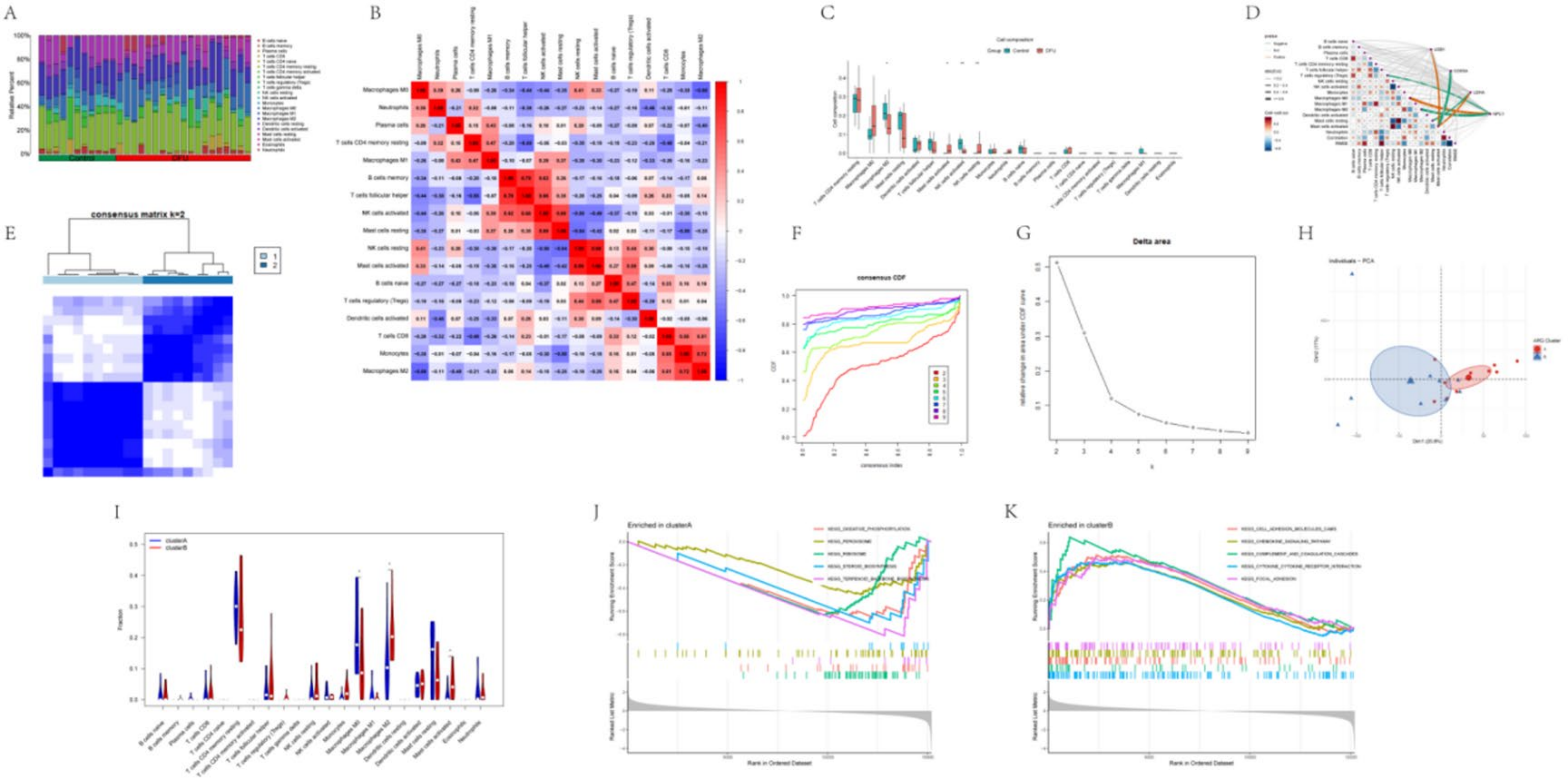
Immune infiltration landscape and identification of hub LRG-related molecular subtypes in DFU. (**A**) Stacked bar plots showing relative proportions of 22 immune cell types. (**B**) Heatmap of pairwise correlations among immune cell subsets. (**C**) Boxplots comparing immune cell fractions between Control and DFU groups. (**D**) Correlation network between hub LRG-related genes and immune cell populations. (**E–G**) Consensus clustering analysis identifying two molecular subtypes (Cluster A and Cluster B) in DFU samples. (**H**) PCA plot showing separation between Cluster A and Cluster B. (**I**) Boxplots showing differences in immune infiltration between Cluster **A** and Cluster **B**. (**J**) GSEA results for Cluster (**A**) (**K**) GSEA results for Cluster (**B**) Statistical significance was defined as *p < 0.05, **p < 0.01, ***p < 0.001 unless otherwise indicated.

To investigate the immune microenvironmental alterations in DFU, we employed the CIBERSORT algorithm to assess the relative proportions of 22 immune cell types between the control and DFU groups, using the merged RNA-seq training cohorts (GSE80178 and GSE134431). The estimated immune cell fractions for all samples are provided in Supplementary Table 3. As shown in Fig. 4A, the immune cell composition differed markedly between the two groups. Correlation analysis among immune cells (Fig. 4B) revealed complex interrelationships, where macrophages M0 were strongly negatively correlated with macrophages M2 and CD4 memory resting T cells, suggesting a dysregulated balance between pro-inflammatory and reparative immune responses. Quantitative comparison demonstrated that Macrophages M2 were significantly reduced in DFU samples compared with controls (Wilcoxon test, *p* = 0.018). In addition, Mast cells activated were significantly increased in DFU tissues (*p* = 0.024). Among NK cell subsets, NK cells activated showed a significant decrease (*p* = 0.009), whereas NK cells resting exhibited a significant increase in DFU samples (*p* = 0.008) (Fig. 4C; Supplementary Table 4). These results indicate a shift in innate immune composition characterized by altered macrophage polarization and NK cell state imbalance in the DFU microenvironment.

Furthermore, using the merged RNA-seq training cohorts (GSE80178 and GSE134431), we constructed a correlation network between hub LRG-related genes and infiltrating immune cell populations, we constructed a correlation network between key genes (*USB1*, *COX5A*, *LDHA*, *NFU1*) and infiltrating immune cell populations (Fig. 4D). NFU1 exhibited a strong negative correlation with pro-inflammatory immune cells such as macrophages M0 and activated dendritic cells, but a positive correlation with anti-inflammatory macrophages M2, indicating its potential role in modulating immune homeostasis in DFU. Similar patterns were observed for *COX5A* and *LDHA*. Collectively, these findings suggest that hub LRG-related genes are intricately involved in immune dysregulation within the DFU microenvironment. Based on the expression profiles of hub LRG-related genes and using the merged training cohorts (GSE80178 and GSE134431), we further performed consensus clustering analysis to identify distinct molecular subtypes in DFU. The optimal clustering stability was achieved when k = 2, dividing the DFU samples into two clusters, designated as Cluster A and Cluster B (Figs. 4E–G). PCA showed a clear separation between the two clusters (Fig. 4H), suggesting distinct transcriptional patterns. Immune infiltration analysis between the two clusters revealed significant differences in specific immune cell populations (Fig. 4I; Supplementary Table 5). Compared with Cluster A, Cluster B exhibited a significantly higher proportion of Macrophages M2 (*p* = 0.013) and a modest but statistically significant increase in Macrophages M0 (*p* = 0.045). In addition, activated mast cells were significantly elevated in Cluster B (*p* = 0.038). The complete statistical results are provided in Supplementary Table 6. The increased abundance of M2 macrophages suggests a distinct immune remodeling state between the two molecular subtypes, potentially reflecting differential tissue repair or immune-regulatory dynamics. The concurrent elevation of M0 macrophages may indicate an altered macrophage differentiation balance within Cluster B.

To further explore the functional characteristics of the two clusters, GSEA was conducted. Cluster A was predominantly enriched in metabolic pathways such as drug metabolism-cytochrome P450, histidine metabolism, neuroactive ligand-receptor interaction, ribosome, and tyrosine metabolism (Fig. 4J), suggesting a prominent metabolic activation status. In contrast, Cluster B was enriched in pathways related to cell adhesion and intracellular trafficking, including adherens junction, endocytosis, focal adhesion, regulation of actin cytoskeleton, and ubiquitin mediated proteolysis (Fig. 4K), indicating enhanced cellular interaction and migration capabilities. These findings indicate that hub LRG-related molecular subtypes in DFU are closely linked to distinct immune microenvironment landscapes and functional states, potentially influencing disease progression and therapeutic responses.

### Validation of hub LRG-related genes and functional characterization of fibroblast subpopulations

**Fig. 5:**
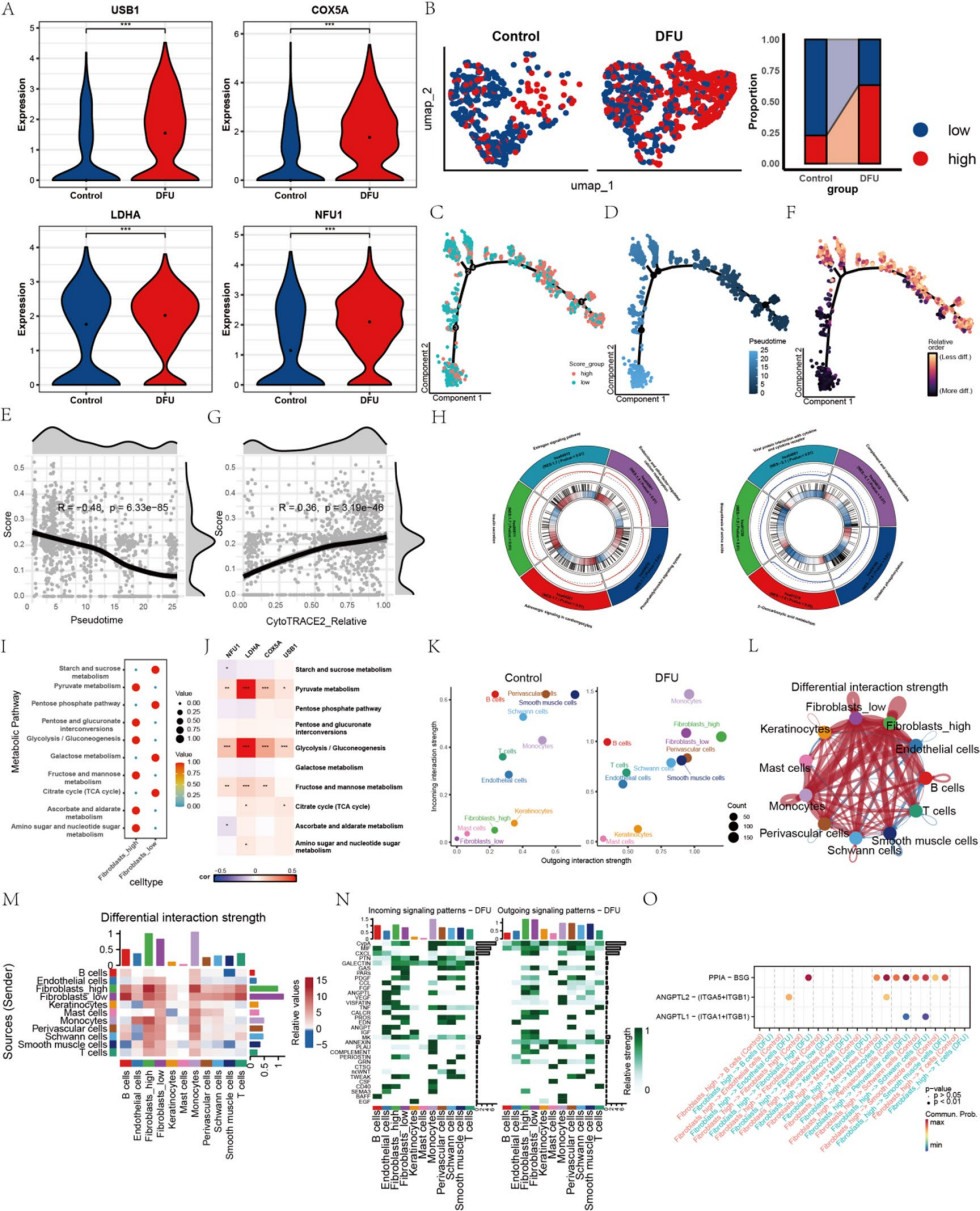
Validation and multi-dimensional characterization of hub LRG-related genes in fibroblast subpopulations. (**A**) Expression patterns of *COX5A*, *LDHA*, *NFU1*, and *USB1* across fibroblasts. (**B**) UMAP visualization and proportional analysis of fibroblasts from Control and DFU groups stratified by LRG activity status (low, blue; high, red). (**C**) Distribution of Fibroblasts_high and Fibroblasts_low groups along the trajectory, showing Fibroblasts_high enrichment at early pseudotime stages. (**D**) Pseudotime trajectory analysis showing the developmental lineage of fibroblasts. (**E**) Negative correlation between hub LRG AUCell scores and pseudotime. (**F**) Distribution of CytoTRACE2 relative differentiation state along the trajectory. (**G**) Positive correlation between hub LRG AUCell scores and CytoTRACE stemness scores. (**H**) GSEA enrichment analysis showing upregulated and downregulated KEGG pathways in Fibroblasts_high. (**I**) Comparison of metabolic pathway activities between Fibroblasts_high and Fibroblasts_low by scMetabolism. (**J**) Correlation heatmap between hub LRGs and metabolic pathways. (**K**) Scatter plot showing differential incoming and outgoing interaction strengths of each cell type in Control and DFU groups. (**L**) Network visualization of differential cell–cell interaction strength in Control and DFU groups. (**M**) Heatmap of differential interaction strength between sender and receiver cell types in DFU relative to Control. (**N**) Heatmaps of altered incoming (left) and outgoing (right) signaling patterns in DFU across major cell types. (**O**) Bubble plot of ANGPTL–integrin ligand–receptor interactions across cell-type pairs. Statistical significance was defined as *p < 0.05, **p < 0.01, ***p < 0.001 unless otherwise indicated.

To validate hub LRGs at the single-cell level, we analyzed the GSE165816 single-cell RNA-sequencing dataset (Fig. 5A) and examined the expression of *COX5A*, *LDHA*, *NFU1*, and *USB1* in fibroblasts from DFU and control groups. All four genes were significantly upregulated in the DFU group (all *p* < 0.001). Based on AUCell scores derived from hub LRG expression, fibroblasts were stratified into Fibroblasts_high and Fibroblasts_low subsets. Fibroblasts_high were predominantly enriched in the DFU group (Fig. 5B).

Trajectory analysis was performed to characterize the differentiation status of fibroblast subsets. Projection of fibroblast subsets onto the developmental trajectory revealed that Fibroblasts_high were predominantly distributed in early pseudotime regions (Fig. 5C), suggesting their association with earlier differentiation states. Consistently, AUCell scores derived from hub LRG expression were negatively correlated with pseudotime (*r* = − 0.48, *p* < 0.001; Fig. 5D–E), indicating that cells with higher hub LRG activity tended to localize at earlier developmental stages. CytoTRACE analysis further supported this observation, demonstrating a positive correlation between AUCell scores and stemness (*r* = 0.36, *p* < 0.001; Fig. 5F–G). Together, these findings are consistent with a less differentiated phenotype in hub LRG–high fibroblasts.

GSEA enrichment analysis based on KEGG pathways revealed that Fibroblasts_high were enriched in glycolysis/gluconeogenesis, the pentose phosphate pathway, and pyruvate metabolism, with relatively reduced oxidative phosphorylation signatures (Fig. 5H). scMetabolism analysis showed increased glycolytic activity and elevated pyruvate-related metabolic flux (Fig. 5I). Hub LRG expression was positively correlated with glycolysis- and pyruvate-associated pathways (Fig. 5J), in agreement with prior enrichment results implicating mitochondrial remodeling, ATP metabolic processes, and hypoxia-responsive pathways. These findings are consistent with a relative shift toward glycolysis-dominant energy metabolism.

CellChat analysis demonstrated that although both subsets were actively involved in intercellular communication, Fibroblasts_high exhibited markedly increased outgoing interaction strength (Fig. 5K), suggesting an enhanced signaling-sending phenotype. Monocytes were identified as the strongest signal recipients and the most prominent interacting cell type with Fibroblasts_high in DFU tissues (Fig. 5L–M), indicating a potential metabolic–inflammatory coupling mechanism. Pathway analysis further revealed strong enrichment of the CypA and ANGPTL signaling pathways in the outgoing signaling profile of Fibroblasts_high, which correspondingly showed increased incoming signaling in monocytes (Fig. 5N). Ligand–receptor analysis confirmed significant activation of the *PPIA-BSG* and *ANGPTL2-ITGA5 + ITGB1* axes in Fibroblasts_high-to-monocyte communication within the DFU group (Fig. 5O).

Collectively, these results suggest that fibroblasts with high hub LRG expression are characterized by an early differentiation state, glycolysis-oriented metabolic features, and enhanced pro-inflammatory signaling toward monocytes, which may contribute to pathological remodeling in DFU tissues.

### Molecular docking and qRT-PCR validation of hub LRG-encoded target proteins in DFU and control samples

**Fig. 6:**
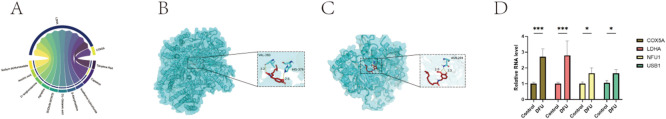
Drug-gene network analysis and molecular docking validation of hub LRGs. (**A**) Chord plot showing candidate compounds targeting hub LRGs identified from the DSigDB database. (**B**) Molecular docking model of capsaicin binding to *COX5A*. (**C**) Molecular docking model of capsaicin binding to *LDHA*. (**D**) qPCR experimental results of four genes expressed in clinical samples. Statistical significance was defined as *p < 0.05, **p < 0.01, ***p < 0.001 unless otherwise indicated.

The identified hub LRGs were further explored for potential therapeutic targeting in DFU. A compound screening analysis was performed using the DSigDB database to identify molecules significantly associated with these genes (Supplementary Table 7). Among the enriched candidates, capsaicin ranked among the top hits and uniquely targeted two hub LRGs (*COX5A* and *LDHA*), whereas most other compounds were linked to a single gene. Based on its multi-gene targeting profile and statistical significance, capsaicin was selected for further evaluation. Molecular docking analysis was subsequently performed to assess the potential binding interactions between capsaicin and *COX5A* and *LDHA* (Figure. 6 A). The docking results showed that capsaicin exhibited strong binding affinities with *COX5A* and *LDHA*, with binding energies of − 7.8 kcal/mol and − 5.7 kcal/mol, respectively (Figs. 6B–C). Generally, a binding energy lower than − 5.0 kcal/mol indicates significant binding strength. These findings suggest that capsaicin may modulate hub LRG activity and provide a novel therapeutic approach for DFU. Six cases each of DFU and normal skin samples were collected and performed qPCR detection of four genes’ expression levels. We found that *COX5A* (*p* < 0.001), *LDHA* (*p* < 0.001), *NFU1* (*p* < 0.014), and *USB1* (*p* < 0.034) were all significantly upregulated in the DFU group (Fig. 6D).

## Disscussion

In this study, we integrated bulk and single-cell transcriptomic analyses to define lactylation-related gene (LRG) signatures in diabetic foot ulcer (DFU) tissues and to identify candidate molecular drivers associated with disease progression. Four hub LRGs—*USB1*, *COX5A*, *LDHA*, and *NFU1*—were consistently upregulated across datasets and exhibited diagnostic potential. These findings extend previous observations that metabolic dysregulation is a central feature of chronic diabetic woundsand suggest that lactylation-associated transcriptional programs may represent an additional regulatory layer within this context^1^.

The identified hub genes converge on mitochondrial function and metabolic homeostasis. *COX5A* and *NFU1* are integral to mitochondrial respiratory chain integrity and iron–sulfur cluster assembly, processes required for efficient oxidative phosphorylation and redox balance^30,31^. *LDHA* catalyzes the reduction of pyruvate to lactate, a metabolic step that becomes dominant under hypoxic or inflammatory conditions^32^. Although *USB1* has been less studied in metabolic disease, emerging data suggest roles in cellular differentiation and immune regulation^33^. Rather than acting independently, the coordinated upregulation of these genes is consistent with a systemic metabolic reconfiguration characteristic of chronic tissue stress.

Single-cell analysis localized these alterations predominantly to fibroblasts. A distinct subset, defined by elevated hub LRG activity (Fibroblasts_high), was enriched in DFU tissues. Trajectory inference indicated that Fibroblasts_high preferentially occupied early pseudotime states, and this positioning was supported by higher stemness scores. These observations suggest that hub LRG–high fibroblasts may reside in a less differentiated state. Given the central role of fibroblast differentiation in extracellular matrix deposition, contractility, and wound resolution, such a shift may influence tissue remodeling dynamics in DFU^34^.

Metabolic profiling provided further mechanistic context. Fibroblasts_high were enriched in glycolysis, the pentose phosphate pathway, and pyruvate metabolism, while exhibiting relatively reduced oxidative phosphorylation signatures. This pattern aligns with earlier enrichment results implicating ATP metabolic processes, mitochondrial remodeling, and hypoxia-responsive pathways. Collectively, these findings indicate a glycolysis-oriented metabolic configuration. From a mechanistic perspective, enhanced glycolytic flux, together with elevated *LDHA* expression, would be expected to increase intracellular lactate availability. Lactate is increasingly recognized not merely as a metabolic end-product but as a signaling metabolite capable of modulating gene expression through histone lactylation and related epigenetic modifications^35^. Within this framework, the metabolic state observed in Fibroblasts_high may provide the biochemical substrate necessary for lactylation-dependent transcriptional regulation. Although direct quantification of lactylation marks was beyond the scope of the present study, the convergence of glycolytic activation, mitochondrial remodeling signatures, and LRG upregulation supports the plausibility of such a mechanism in DFU fibroblasts.

The enrichment of DNA replication and repair pathways in *NFU1*- and *USB1*-associated signatures should be interpreted within the context of metabolic stress. Chronic hyperglycemia and sustained glycolytic activation promote mitochondrial dysfunction and redox imbalance, and persistent oxidative stress is known to induce replication stress and DNA damage, thereby activating genomic maintenance programs^36^. In chronic wounds, such activation likely reflects stress adaptation rather than effective regeneration. Given its role in iron–sulfur cluster assembly and mitochondrial integrity, *NFU1* provides a mechanistic link between metabolic disturbance and DNA repair signaling. Accordingly, the observed enrichment of DNA repair pathways is consistent with sustained metabolic stress in DFU fibroblasts and may contribute to impaired wound resolution.

Beyond cell-intrinsic metabolism, intercellular communication analysis revealed that Fibroblasts_high exhibited enhanced outgoing signaling strength, particularly toward monocytes. Notably, activation of the *PPIA–BSG* and *ANGPTL2–ITGA5 + ITGB1* axes was observed in DFU tissues. *PPIA* (*Cyclophilin A*) functions as a stress-responsive secreted mediator capable of amplifying inflammatory signaling through *CD147/BSG*, while *ANGPTL2* has been implicated in chronic inflammation and integrin-dependent extracellular matrix remodeling^37,38^. The coupling of glycolysis-oriented metabolism with strengthened fibroblast–monocyte communication suggests a coordinated program in which metabolically reprogrammed fibroblasts may contribute to sustained inflammatory signaling. Emerging studies indicate that lactate and lactylation can influence immune cell activation and polarization, raising the possibility that metabolic reconfiguration in fibroblasts may indirectly shape immune responses in the DFU microenvironment^39^.

From a translational perspective, molecular docking analysis indicated favorable binding interactions between capsaicin and selected hub LRG proteins, particularly *COX5A* and *LDHA*. Capsaicin has been reported to exert anti-inflammatory and metabolic regulatory effects in various disease models^40^. Although docking results alone cannot establish functional efficacy, these findings raise the possibility that modulation of metabolic enzymes involved in lactate production and mitochondrial function may represent a potential therapeutic strategy in DFU. Experimental validation will be necessary to determine whether capsaicin or related compounds can influence fibroblast metabolic states and downstream inflammatory signaling in vivo.

Several limitations warrant consideration. Our conclusions are primarily based on transcriptomic inference, and validation at the protein and post-translational modification levels, particularly with respect to histone lactylation, will be essential to substantiate mechanistic interpretations. In addition, although multiple datasets were integrated, sample size constraints may affect generalizability. Importantly, chronic DFU wounds are frequently colonized by microbial biofilms, which may contribute additional lactate to the wound microenvironment. The present study does not distinguish between host- and bacteria-derived lactate, and future investigations integrating microbiome and metabolomic profiling will be required to clarify their relative contributions to lactylation-associated regulation. Functional studies will also be necessary to determine whether targeting hub LRGs or associated signaling axes can modulate fibroblast differentiation, immune crosstalk, and wound repair outcomes.

In summary, our findings delineate a fibroblast-centered metabolic program in DFU characterized by reduced differentiation signatures, enhanced glycolytic flux, and strengthened pro-inflammatory intercellular communication. While further validation is required, these data support a model in which lactate-associated metabolic reprogramming may intersect with immune signaling to influence pathological remodeling in chronic diabetic wounds.

## Conclusion

In conclusion, our integrative analyses identify a lactate-associated molecular signature in DFU characterized by glycolysis-oriented metabolic reprogramming, reduced differentiation features in fibroblasts, and enhanced pro-inflammatory intercellular communication. Hub LRG–high fibroblasts may contribute to pathological tissue remodeling through coordinated metabolic and immune signaling mechanisms. The identified hub LRGs—*USB1*, *COX5A*, *LDHA*, and *NFU1*—demonstrate diagnostic potential and represent candidate molecular nodes within this metabolic–inflammatory network. Molecular docking further suggests that capsaicin may interact with selected hub proteins, highlighting a potential avenue for therapeutic exploration. While further validation is required, these findings provide a mechanistic framework linking lactate-associated pathways to DFU progression.

## Supplementary Information

Below is the link to the electronic supplementary material.

Supplementary Material 1

Supplementary Material 2

Supplementary Material 3

Supplementary Material 4

Supplementary Material 5

Supplementary Material 6

Supplementary Material 7

Supplementary Material 8

Supplementary Material 9

## Data Availability

The scRNA-seq dataset used in this study, GSE165816, comprising 10 DFU samples and 19 control foot skin tissue samples, is publicly available in the Gene Expression Omnibus (GEO) database and can be accessed through the following link: https://www.ncbi.nlm.nih.gov/geo/query/acc.cgi? acc=GSE165816. In addition, four bulk RNA sequencing datasets were used for machine learning analyses: GSE80178 and GSE134431 were used as the training cohort, while GSE199939 and GSE68183 served as the validation cohort. All datasets are publicly available in the GEO database.

## References

[1] Senneville, É. et al. IWGDF/IDSA guidelines on the diagnosis and treatment of diabetes-related foot infections (IWGDF/IDSA 2023). *Diabetes Metab. Res. Rev.* **40** (3), e3687 (2024). 37779323 10.1002/dmrr.3687

[2] Jeffcoate, W. J., Vileikyte, L., Boyko, E. J., Armstrong, D. G. & Boulton, A. J. M. Current Challenges and Opportunities in the Prevention and Management of Diabetic Foot Ulcers. *Diabetes Care*. **41** (4), 645–652 (2018). 29559450 10.2337/dc17-1836

[3] Hinchliffe, R. J. et al. IWGDF guidance on the diagnosis, prognosis and management of peripheral artery disease in patients with foot ulcers in diabetes. *Diabetes Metab. Res. Rev.* **32** (Suppl 1), 37–44 (2016). 26332424 10.1002/dmrr.2698

[4] Zhang, D. et al. Metabolic regulation of gene expression by histone lactylation. *Nature* **574** (7779), 575–580 (2019). 31645732 10.1038/s41586-019-1678-1 PMC6818755

[5] Wang, J., Wang, Z., Wang, Q., Li, X. & Guo, Y. Ubiquitous protein lactylation in health and diseases. *Cell. Mol. Biol. Lett.* **29** (1), 23 (2024). 38317138 10.1186/s11658-024-00541-5 PMC10845568

[6] Chirumbolo, S., Bertossi, D. & Magistretti, P. Insights on the role of L-lactate as a signaling molecule in skin aging. *Biogerontology* **24** (5), 709–726 (2023). 36708434 10.1007/s10522-023-10018-1 PMC9883612

[7] Hao, Y. et al. Integrated analysis of multimodal single-cell data. *Cell* **184** (13), 3573–3587e3529 (2021). 34062119 10.1016/j.cell.2021.04.048 PMC8238499

[8] Theocharidis, G. et al. Single cell transcriptomic landscape of diabetic foot ulcers. *Nat. Commun.* **13** (1), 181 (2022). 35013299 10.1038/s41467-021-27801-8 PMC8748704

[9] Sreedhar, A., Aguilera-Aguirre, L. & Singh, K. K. Mitochondria in skin health, aging, and disease. *Cell. Death Dis.* **11** (6), 444 (2020). 32518230 10.1038/s41419-020-2649-z PMC7283348

[10] Jiang, N. et al. Comprehensive transcriptomic analysis of immune-related genes in diabetic foot ulcers: New insights into mechanisms and therapeutic targets. *Int. Immunopharmacol.* **139**, 112638 (2024). 39079197 10.1016/j.intimp.2024.112638

[11] Ramirez, H. A. et al. Staphylococcus aureus Triggers Induction of miR-15B-5P to Diminish DNA Repair and Deregulate Inflammatory Response in Diabetic Foot Ulcers. *J. Invest. Dermatol.* **138** (5), 1187–1196 (2018). 29273315 10.1016/j.jid.2017.11.038 PMC6358418

[12] Sawaya, A. P. et al. Deregulated immune cell recruitment orchestrated by FOXM1 impairs human diabetic wound healing. *Nat. Commun.* **11** (1), 4678 (2020). 32938916 10.1038/s41467-020-18276-0 PMC7495445

[13] Shi, Y. et al. Integrating Bulk RNA and Single-Cell RNA Sequencing Identifies and Validates Lactylation-Related Signatures for Intervertebral Disc Degeneration. *J. Cell. Mol. Med.* **28** (23), e70262 (2024). 39636180 10.1111/jcmm.70262 PMC11619158

[14] Park, P. G. et al. Single-cell transcriptomics in a child with coenzyme Q10 nephropathy: potential of single-cell RNA sequencing in pediatric kidney disease. *Pediatr. Nephrol.* **40** (5), 1653–1662 (2025). 39805995 10.1007/s00467-024-06611-2 PMC11946986

[15] Jia, Y. et al. Metabolic Heterogeneity of Tumor Cells and its Impact on Colon Cancer Metastasis: Insights from Single-Cell and Bulk Transcriptome Analyses. *J. Cancer*. **15** (13), 4175–4196 (2024). 38947396 10.7150/jca.94630 PMC11212087

[16] McGinnis, C. S., Murrow, L. M. & Gartner, Z. J. DoubletFinder: Doublet Detection in Single-Cell RNA Sequencing Data Using Artificial Nearest Neighbors. *Cell. Syst.* **8** (4), 329–337e324 (2019). 30954475 10.1016/j.cels.2019.03.003 PMC6853612

[17] Aran, D. et al. Reference-based analysis of lung single-cell sequencing reveals a transitional profibrotic macrophage. *Nat. Immunol.* **20** (2), 163–172 (2019). 30643263 10.1038/s41590-018-0276-y PMC6340744

[18] Aibar, S. et al. SCENIC: single-cell regulatory network inference and clustering. *Nat. Methods*. **14** (11), 1083–1086 (2017). 28991892 10.1038/nmeth.4463 PMC5937676

[19] Morabito, S., Reese, F., Rahimzadeh, N., Miyoshi, E. & Swarup, V. hdWGCNA identifies co-expression networks in high-dimensional transcriptomics data. *Cell. Rep. Methods*. **3** (6), 100498 (2023). 37426759 10.1016/j.crmeth.2023.100498 PMC10326379

[20] Skinnider, M. A. et al. Cell type prioritization in single-cell data. *Nat. Biotechnol.* **39** (1), 30–34 (2021). 32690972 10.1038/s41587-020-0605-1 PMC7610525

[21] Jin, S. et al. Inference and analysis of cell-cell communication using CellChat. *Nat. Commun.* **12** (1), 1088 (2021). 33597522 10.1038/s41467-021-21246-9 PMC7889871

[22] Gulati, G. S. et al. Single-cell transcriptional diversity is a hallmark of developmental potential. *Science* **367** (6476), 405–411 (2020). 31974247 10.1126/science.aax0249 PMC7694873

[23] Wu, Y. et al. Spatiotemporal Immune Landscape of Colorectal Cancer Liver Metastasis at Single-Cell Level. *Cancer Discov*. **12** (1), 134–153 (2022). 34417225 10.1158/2159-8290.CD-21-0316

[24] Wu, T. et al. clusterProfiler 4.0: A universal enrichment tool for interpreting omics data. *Innov. (Camb)*. **2** (3), 100141 (2021). 10.1016/j.xinn.2021.100141 PMC8454663 34557778

[25] Subramanian, A. et al. Gene set enrichment analysis: a knowledge-based approach for interpreting genome-wide expression profiles. *Proc. Natl. Acad. Sci. U S A*. **102** (43), 15545–15550 (2005). 16199517 10.1073/pnas.0506580102 PMC1239896

[26] Hänzelmann, S., Castelo, R. & Guinney, J. GSVA: gene set variation analysis for microarray and RNA-seq data. *BMC Bioinform.* **14**, 7 (2013). 10.1186/1471-2105-14-7 PMC3618321 23323831

[27] Newman, A. M. et al. Robust enumeration of cell subsets from tissue expression profiles. *Nat. Methods*. **12** (5), 453–457 (2015). 25822800 10.1038/nmeth.3337 PMC4739640

[28] Wilkerson, M. D. & Hayes, D. N. ConsensusClusterPlus: a class discovery tool with confidence assessments and item tracking. *Bioinformatics* **26** (12), 1572–1573 (2010). 20427518 10.1093/bioinformatics/btq170 PMC2881355

[29] Zhang, Y. et al. Identification of 3 key genes as novel diagnostic and therapeutic targets for OA and COVID-19. *Front. Immunol.* **14**, 1167639 (2023). 37283761 10.3389/fimmu.2023.1167639 PMC10239847

[30] Kadenbach, B. & Hüttemann, M. The subunit composition and function of mammalian cytochrome c oxidase. *Mitochondrion* **24**, 64–76 (2015). 26190566 10.1016/j.mito.2015.07.002

[31] Kaiyrzhanov, R. et al. Phenotypic continuum of NFU1-related disorders. *Ann. Clin. Transl Neurol.* **9** (12), 2025–2035 (2022). 36256512 10.1002/acn3.51679 PMC9735368

[32] Fang, L., Yu, Z., Qian, X., Fang, H. & Wang, Y. LDHA exacerbates myocardial ischemia-reperfusion injury through inducing NLRP3 lactylation. *BMC Cardiovasc. Disord*. **24** (1), 651 (2024). 39548367 10.1186/s12872-024-04251-w PMC11568565

[33] Fazi, F. et al. A minicircuitry comprised of microRNA-223 and transcription factors NFI-A and C/EBPalpha regulates human granulopoiesis. *Cell* **123** (5), 819–831 (2005). 16325577 10.1016/j.cell.2005.09.023

[34] Talbott, H. E., Mascharak, S., Griffin, M., Wan, D. C. & Longaker, M. T. Wound healing, fibroblast heterogeneity, and fibrosis. *Cell. Stem Cell.* **29** (8), 1161–1180 (2022). 35931028 10.1016/j.stem.2022.07.006 PMC9357250

[35] Xu, B., Liu, Y., Li, N. & Geng, Q. Lactate and lactylation in macrophage metabolic reprogramming: current progress and outstanding issues. *Front. Immunol.* **15**, 1395786 (2024). 38835758 10.3389/fimmu.2024.1395786 PMC11148263

[36] Almalki, S., Salama, M., Taylor, M. J., Ahmed, Z. & Tuxworth, R. I. C9orf72-related amyotrophic lateral sclerosis-frontotemporal dementia and links to the DNA damage response: a systematic review. *Front. Mol. Neurosci.* **18**, 1671906 (2025). 41341655 10.3389/fnmol.2025.1671906 PMC12669223

[37] Yurchenko, V., Constant, S., Eisenmesser, E. & Bukrinsky, M. Cyclophilin-CD147 interactions: a new target for anti-inflammatory therapeutics. *Clin. Exp. Immunol.* **160** (3), 305–317 (2010). 20345978 10.1111/j.1365-2249.2010.04115.x PMC2883100

[38] Kadomatsu, T., Endo, M., Miyata, K. & Oike, Y. Diverse roles of ANGPTL2 in physiology and pathophysiology. *Trends Endocrinol. Metab.* **25** (5), 245–254 (2014). 24746520 10.1016/j.tem.2014.03.012

[39] Lin, J. & Ren, J. Lactate-induced lactylation and cardiometabolic diseases: From epigenetic regulation to therapeutics. *Biochim. Biophys. Acta Mol. Basis Dis.* **1870** (6), 167247 (2024). 38762059 10.1016/j.bbadis.2024.167247

[40] Sharma, S. K., Vij, A. S. & Sharma, M. Mechanisms and clinical uses of capsaicin. *Eur. J. Pharmacol.* **720** (1–3), 55–62 (2013). 24211679 10.1016/j.ejphar.2013.10.053

