# Supplementary material for: Integrating bulk and single-cell RNA sequencing identifies and validates lactylation-related signatures in diabetic foot ulcers
Source: Sci Rep. 2026 Apr 27;16:19471. doi: 10.1038/s41598-026-49753-z (PMC13287707; doi:10.1038/s41598-026-49753-z)
Supplement: Supplementary file 3 — Supplementary Material 3 [file 41598_2026_49753_MOESM3_ESM.docx]

Supplementary Table 1 Summary of Hyperparameter Tuning and Optimal Settings

| GSM ID | Tissue | Phenotype | Group Label | Inclusion Rationale |
| --- | --- | --- | --- | --- |
| GSM5050523 | Foot skin | Healing DFU | DFU_foot_skin | DFU |
| GSM5050527 | Foot skin | Healing DFU | DFU_foot_skin | DFU |
| GSM5050531 | Foot skin | Healing DFU | DFU_foot_skin | DFU |
| GSM5050532 | Foot skin | Healing DFU | DFU_foot_skin | DFU |
| GSM5050539 | Foot skin | Healing DFU | DFU_foot_skin | DFU |
| GSM5050547 | Foot skin | Healing DFU | DFU_foot_skin | DFU |
| GSM5050566 | Foot skin | Healing DFU | DFU_foot_skin | DFU |
| GSM5050569 | Foot skin | Healing DFU | DFU_foot_skin | DFU |
| GSM5050573 | Foot skin | Healing DFU | DFU_foot_skin | DFU |
| GSM5050530 | Foot skin | Non-healing DFU | DFU_foot_skin | DFU |
| GSM5050533 | Foot skin | Non-healing DFU | DFU_foot_skin | DFU |
| GSM5050557 | Foot skin | Non-healing DFU | DFU_foot_skin | DFU |
| GSM5050558 | Foot skin | Non-healing DFU | DFU_foot_skin | DFU |
| GSM5050563 | Foot skin | Non-healing DFU | DFU_foot_skin | DFU |
| GSM5050534 | Foot skin | Healthy non-diabetic | Control_foot_skin | control |
| GSM5050538 | Foot skin | Healthy non-diabetic | Control_foot_skin | control |
| GSM5050540 | Foot skin | Healthy non-diabetic | Control_foot_skin | control |
| GSM5050548 | Foot skin | Healthy non-diabetic | Control_foot_skin | control |
| GSM5050552 | Foot skin | Healthy non-diabetic | Control_foot_skin | control |
| GSM5050555 | Foot skin | Healthy non-diabetic | Control_foot_skin | control |
| GSM5050556 | Foot skin | Healthy non-diabetic | Control_foot_skin | control |
| GSM5050564 | Foot skin | Healthy non-diabetic | Control_foot_skin | control |
| GSM5050567 | Foot skin | Healthy non-diabetic | Control_foot_skin | control |
| GSM5050568 | Foot skin | Healthy non-diabetic | Control_foot_skin | control |
| GSM5050574 | Foot skin | Healthy non-diabetic | Control_foot_skin | control |
| GSM5050522 | Foot skin | Diabetic without DFU | Control_foot_skin | control |
| GSM5050524 | Foot skin | Diabetic without DFU | Control_foot_skin | control |
| GSM5050525 | Foot skin | Diabetic without DFU | Control_foot_skin | control |
| GSM5050526 | Foot skin | Diabetic without DFU | Control_foot_skin | control |
| GSM5050529 | Foot skin | Diabetic without DFU | Control_foot_skin | control |
| GSM5050562 | Foot skin | Diabetic without DFU | Control_foot_skin | control |
| GSM5050565 | Foot skin | Diabetic without DFU | Control_foot_skin | control |
| GSM5050570 | Foot skin | Diabetic without DFU | Control_foot_skin | control |
