# Supplementary material for: Integrating bulk and single-cell RNA sequencing identifies and validates lactylation-related signatures in diabetic foot ulcers
Source: Sci Rep. 2026 Apr 27;16:19471. doi: 10.1038/s41598-026-49753-z (PMC13287707; doi:10.1038/s41598-026-49753-z)
Supplement: Supplementary file 4 — Supplementary Material 4 [file 41598_2026_49753_MOESM4_ESM.docx]

Supplementary Table 2 Hub genes in DFU_Fibroblasts

| gene name | module |
| --- | --- |
| HSD3B7 | red module |
| ZNF385A | red module |
| PRG4 | red module |
| TPPP3 | red module |
| UAP1 | red module |
| UGDH | red module |
| SDC2 | red module |
| EMP1 | red module |
| TAGLN2 | red module |
| AK1 | red module |
| COX5A | red module |
| CDC42 | red module |
| FABP3 | red module |
| AQP1 | red module |
| KLF4 | red module |
| CDA | red module |
| HAS1 | red module |
| UGP2 | red module |
| GLUD1 | red module |
| GFPT2 | red module |
| ANXA1 | red module |
| NFU1 | red module |
| TUBA1C | red module |
| TRIM47 | red module |
| LDHA | red module |
| FSCN1 | red module |
| ACAT2 | red module |
| STX12 | red module |
| CD151 | red module |
| ARPC5 | red module |
| FBN1 | brown module |
| TNXB | brown module |
| SCARA5 | brown module |
| PCOLCE2 | brown module |
| ELN | brown module |
| CD248 | brown module |
| FSTL1 | brown module |
| FBLN2 | brown module |
| CLEC3B | brown module |
| SEMA3C | brown module |
| FDX2 | brown module |
| PI16 | brown module |
| FN1 | brown module |
| CD55 | brown module |
| PTGIS | brown module |
| AHNAK | brown module |
| ADAMTS5 | brown module |
| TIMP3 | brown module |
| EDIL3 | brown module |
| EMILIN2 | brown module |
| LOXL1 | brown module |
| MRC2 | brown module |
| CREB5 | brown module |
| VCAN | brown module |
| ISLR | brown module |
| ADAMTSL4 | brown module |
| LGR4 | brown module |
| USB1 | brown module |
| TRIO | brown module |
| CADM3 | brown module |
