# Supplementary figures and images for: Integrating bulk and single-cell RNA sequencing identifies and validates lactylation-related signatures in diabetic foot ulcers
Source: Sci Rep. 2026 Apr 27;16:19471. doi: 10.1038/s41598-026-49753-z (PMC13287707; doi:10.1038/s41598-026-49753-z)

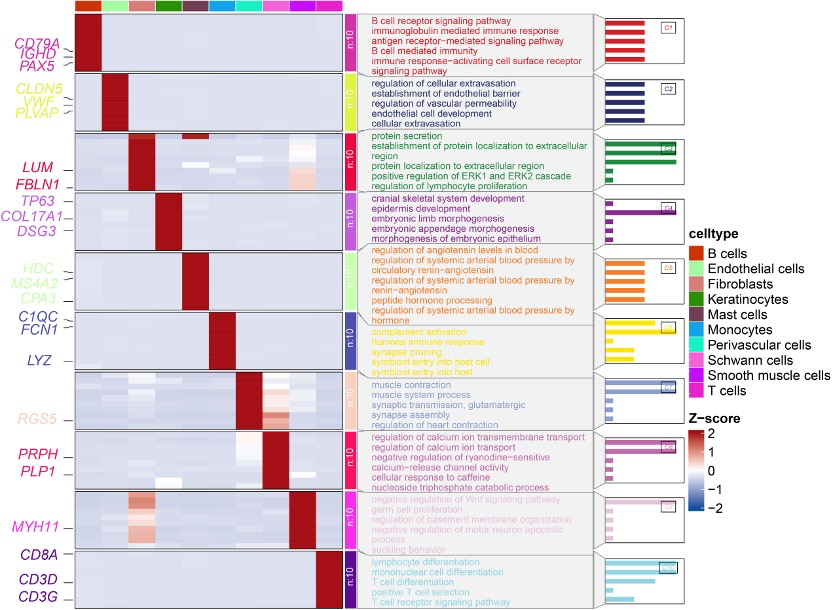

Supplement: Supplementary file 7 — Supplementary Material 7 [file 41598_2026_49753_MOESM7_ESM.tiff]

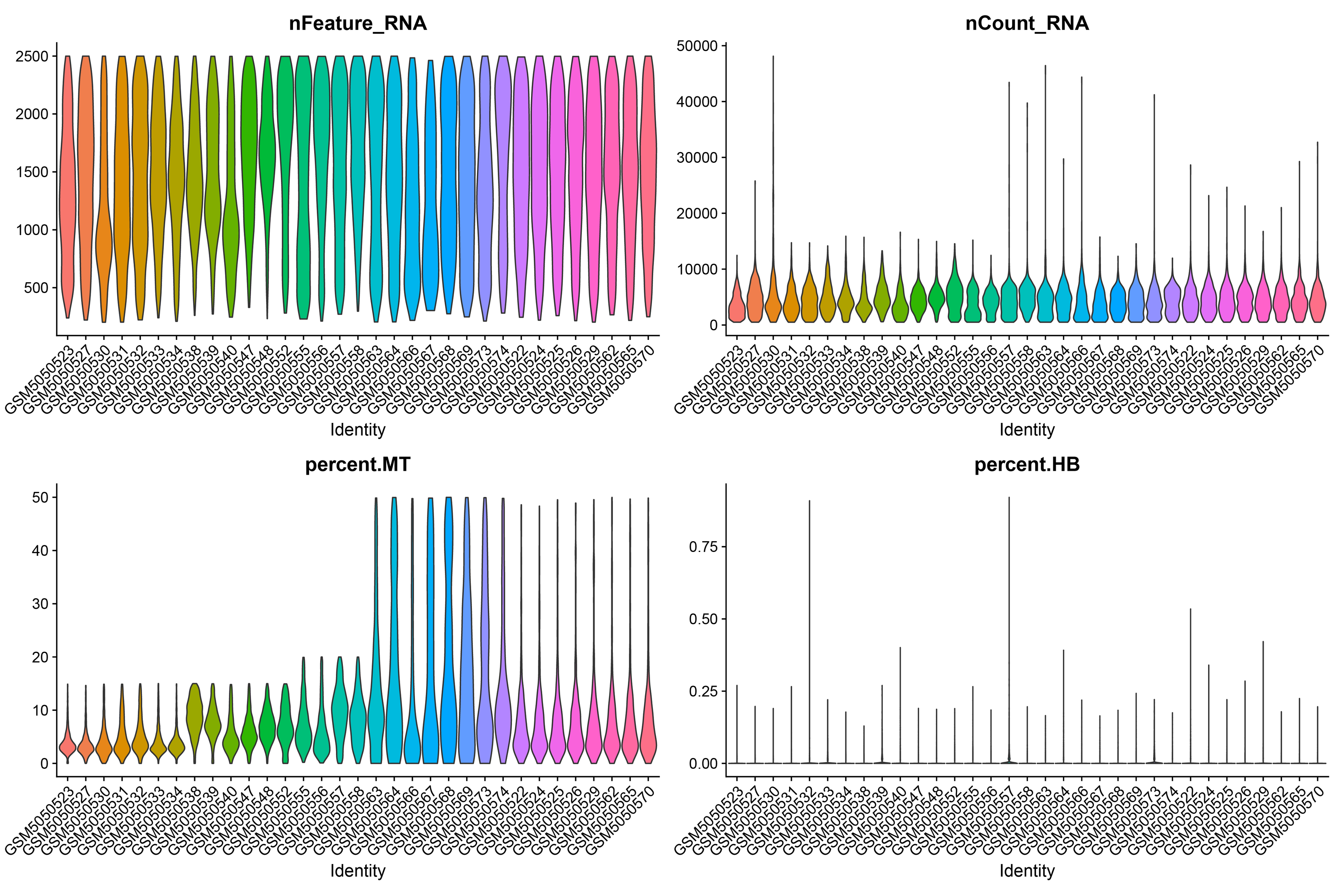

Supplement: Supplementary file 8 — Supplementary Material 8 [file 41598_2026_49753_MOESM8_ESM.tiff]
