# Supplementary material for: Integrating bulk and single-cell RNA sequencing identifies and validates lactylation-related signatures in diabetic foot ulcers
Source: Sci Rep. 2026 Apr 27;16:19471. doi: 10.1038/s41598-026-49753-z (PMC13287707; doi:10.1038/s41598-026-49753-z)
Supplement: Supplementary file 9 — Supplementary Material 9 [file 41598_2026_49753_MOESM9_ESM.docx]

**Table_S6. Compounds significantly enriched for hub lactate-related genes (LRGs) identified using the DSigDB database**

| **Compound** | **Associated hub gene(s)** | **Count** | **Fold Enrichment** | **z-score** | **Adjusted P value** |
| --- | --- | --- | --- | --- | --- |
| Toluylene Red | LDHA | 1 | 491.175 | 22.12 | 0.04179 |
| Capsaicin | COX5A, LDHA | 2 | 26.69 | 7.10 | 0.04179 |
| Isoetharine hydrochloride | LDHA | 1 | 446.52 | 21.09 | 0.04179 |
| β-Alanyl-L-histidine | LDHA | 1 | 409.31 | 20.19 | 0.04179 |
| DL-Glutamic acid | LDHA | 1 | 409.31 | 20.19 | 0.04179 |
| Sodium nitrite | LDHA | 1 | 409.31 | 20.19 | 0.04179 |
| Paeoniflorin | LDHA | 1 | 327.45 | 18.05 | 0.04179 |
| Dantrolene sodium | LDHA | 1 | 306.98 | 17.47 | 0.04179 |
